# *ψ*-Contraction and $(\phi ,\varphi )$-contraction in Menger probabilistic metric space

**DOI:** 10.1186/s40064-016-1795-3

**Published:** 2016-02-29

**Authors:** Pengcheng Ma, Jinyu Guan, Yanxia Tang, Yongchun Xu, Yongfu Su

**Affiliations:** Department of Mathematics, College of Science, Hebei North University, Zhangjiakou, 075000 People’s Republic of China; Department of Mathematics, Tianjin Polytechnic University, Tianjin, 300387 People’s Republic of China

**Keywords:** Probabilistic metric spaces, $(\phi , \varphi )$-Contraction, $\psi$-Contraction, Contraction mapping principle

## Abstract

The purpose of this paper is to present the definition of $(\phi ,\varphi )$-contractive mapping and to discuss the relation of $\psi$-contractive mappings and $(\phi ,\varphi )$-contractive mappings. Furthermore, the generalized $(\phi ,\varphi )$-contraction mapping principle has been proved without the uniqueness condition. Meanwhile, the generalized $\psi$-contraction mapping principle has been obtained by using an ingenious method.

## Introduction and preliminaries

Sometimes, it is found appropriate to assign the average of several measurements as a measure to ascertain the distance between two points. Inspired from this line of thinking, Menger (1942, 1951) introduced the notion of probabilistic metric spaces as a generalization of metric spaces. In fact, he replaced the distance function *d*(*x*, *y*) with a distribution function $F_{x,y}:X\times X\rightarrow R$ wherein for any number *t*, the value $F_{x,y}(t)$ describes the probability that the distance between *x* and *y* is less than *t*. In fact the study of such spaces received an impetus with the pioneering work of Schweizer and Sklar (1983). The theory of probabilistic metric spaces is of paramount importance in random functional analysis especially due to its extensive applications in random differential as well as random integral equations (Chang et al. 1994). Sehgal and Bharucha-Reid (1972; Sehgal 1966) established fixed point theorems in probabilistic metric spaces (for short, PM-spaces). Indeed, by using the notion of probabilistic qcontraction, they proved a unique fixed point result, which is an extension of the celebrated Banach contraction principle (Banach 1922). For the interested reader, a comprehensive study of fixed point theory in the probabilistic metric setting can be found in the book of Hadǐić and Pap (2001), see also Van An et al. (2014) for further discussion on generalizations of metric fixed point theory. Recently, Choudhury and Das (2008) gave a generalized unique fixed point theorem by using an altering distance function, which was originally introduced by Khan et al. (1984). For other results in this direction, we refer to Chauhan et al. (2013, 2014a, b, c, d), Choudhury et al. (2008), Choudhury and Das (2009), Ćirić (1975), Gajić and Rakoćević (2007), Mihet (2009), Dutta et al. (2009), Hadzi and Pap (2001), Kutbi et al. (2015). In particular, Dutta et al. (2009) defined nonlinear generalized contractive type mappings involving altering distances (say, $\psi$-contractive mappings) in Menger PM-spaces and proved their theorem for such kind of mappings in the setting of *G*-complete Menger PM-spaces. On contributing to this study, In 2015, Marwan Amin Kutbi et al. weakened the notion of $\psi$-contractive mapping and establish some fixed point theorems in *G*-complete and *M*-complete Menger PM-spaces, besides discussing some related results and illustrative examples.

Next we shall recall some well-known definitions and results in the theory of probabilistic metric spaces which are used later on in this paper. For more details, we refer the reader to Chauhan et al. (2014a, b), Kutbi et al. (2015), Xu et al. (2015a, b), Chauhan and Pant (2014), Su and Zhang (2014), Su et al. (2015).

### **Definition 1**

A triangular norm (shorter *T*-norm) is a binary operation *T* on [0, 1] which satisfies the following conditions:

- *T* is associative and commutative;
- *T* is continuous;
- $$T(a,1)=a$$ for all $$a\in [0,1];$$
- $T(a,b)\le T(c,d)$ whenever $a \le c$ and $b \le d$ for each $a, b, c, d \in [0,1].$

The following are the four basic *T*-norms:

$$\begin{aligned} T_1(a,b) &= \max (a+b-1,0);\\ T_2(a,b) &= a\cdot b;\\ T_3(a,b) &= {\left\{ \begin{array}{ll} \frac{ab}{a+b-ab}, \quad ab\ne 0\,\\ 0, \quad ab=0; \end{array}\right. }\\ T_4(a,b) &= \min (a,b). \end{aligned}$$

It is easy to check, the above four *T*-norms have the following relations:

$$T_1(a,b)\le T_2(a,b)\le T_3(a,b)\le T_4(a,b),$$

for any $$a,b \in [0,1]$$.

### **Definition 2**

A function $F(t): (-\infty ,+\infty )\rightarrow [0, 1]$ is called a distance distribution function if it is non-decreasing and left-continuous with $\lim _{t\rightarrow -\infty }F(t)=0, \quad \lim _{t\rightarrow +\infty }F(t)=1$. and $F(0)=0$. The set of all distance distribution functions is denoted by $D^+$. A special distance distribution function is given by

$$H(t)={\left\{ \begin{array}{ll} 0, \quad t\le 0,\\ 1, \quad t>0. \end{array}\right. }$$

### **Definition 3**

A Menger probabilistic metric space is a triple (*E*, *F*, *T*) where *E* is a nonempty set, *T* is a continuous *t*-norm and *F* is a mapping from $E\times E$ into $D^+$ such that, if $F_{x,y}$ denotes the value of *F* at the pair (*x*, *y*), the following conditions hold:

- (MPM-1) $F_{x,y}(t)=H(t)$ if and only if $x=y$;
- (MPM-2) $F_{x,y}(t)=F_{y,x}(t)$ for all $x,y \in E$ and $t \in (-\infty ,+\infty);$
- (MPM-3) $F_{x,y}(t+s)\ge T (F_{x,z}(t),F_{z,y}(s))$ for all $x,y,z \in E$ and $t>0, s>0.$

### **Definition 4**

(Kutbi et al. 2015) Let (*E*, *F*, *T*) be a Menger probabilistic metric space.

1. A sequence $\{x_n\}$ in *E* is said to converge to $x\in E$ if for any given $\varepsilon > 0$ and $\lambda >0$, there exists a positive integer $N = N(\varepsilon , \lambda )$ such that $F_{x_n,x}(\varepsilon )>1-\lambda$ whenever $n>N$.
2. A sequence $\{x_n\}$ in *E* is called a Cauchy sequence if for any $\varepsilon >0$ and $\lambda >0$, there exists a positive integer $N = N(\varepsilon ,\lambda )$ such that $F_{x_n,x_m}(\varepsilon )>1-\lambda$, whenever $n,m >N$.
3. (*E*, *F*, *T*) is said to be *M*-complete if each Cauchy sequence in *E* converges to some point in *E*.
4. A sequence $\{x_n\}$ in *E* is called a *G*-Cauchy sequence if $\lim _{n\rightarrow \infty }F_{x_n,x_{n+m}}(t)=0$ for any given positive integer *m* and $t>0$.
5. (*E*, *F*, *T*) is said to be *G*-complete if each *G*-Cauchy sequence is convergent in *E*.

### *Example*

Let $x_n=\sum _{i=1}^{n}\frac{1}{i}, \ n=1, 2, 3, \ldots$. It is easy to show, for any given *m*, that

$$|x_n-x_{n+m}|=\sum _{i=n+1}^{n+m}\frac{1}{i}\le \frac{m}{n+1}\rightarrow 0$$

as $n\rightarrow \infty$. Hence $\{x_n\}$ is a *G*-Cauchy sequence. But it is not a Cauchy sequence, since $x_n$ does not converge.

### **Definition 5**

(Kutbi et al. 2015) A function $\phi :R^+ \rightarrow R^+$ is said to be a $\phi$-function if it satisfies the following conditions:

(i) $\phi (t) =0$ if and only if $t = 0$;
(ii) $\phi (t)$ is strictly increasing and $\phi (t)\rightarrow \infty$ as $t\rightarrow \infty$;
(iii) $\phi (t)$ is left continuous in $(0,+\infty )$;
(iv) $\phi (t)$ is continuous at 0.

In the sequel, the class of all $\phi$-functions will be denoted by $\Phi$. We denote by $\Psi$ the class of all continuous non-decreasing functions $\psi : R^+ \rightarrow R^+$ such that $\psi (0) = 0$ and $\psi ^n(a_n)\rightarrow 0$, whenever $a_n\rightarrow 0$, as $n\rightarrow \infty$.

Kutbi et al. (2015) proved the two generalized contraction mapping principles for the following so-called $\psi$-contractive mapping *T* from a Menger probabilistic metric space (*E*, *F*, *T*) into it-self:

$$\frac{1}{F_{fx,fy}(\phi (ct))}-1\le \psi \left( \frac{1}{F_{x,y}(\phi (t))}-1\right) , \quad \forall t>0, \; \forall x,y \in E$$

where $c \in (0,1)$ and $\psi (t), \phi (t)$ are two functions with the suitable conditions. In so-called *M*-complete Menger probabilistic spaces, they have proved a generalized $\psi$-contraction mapping principle provided that *F* is triangular:

$$\frac{1}{F_{x,y}(t)}-1\le \frac{1}{F_{x,z}(t)}-1+\frac{1}{F_{z,y}(t)}-1,$$

for every $x, y, z \in E$ and each $t > 0$.

The purpose of this paper is to present the definition of $(\phi ,\varphi )$-contractive mapping and to discuss the relation of $\psi$-contractive mappings and $(\phi ,\varphi )$-contractive mappings. Furthermore, the generalized $(\phi ,\varphi )$-contraction mapping principle has been proved without the uniqueness condition. Meanwhile, the generalized $\psi$-contraction mapping principle has been obtained by using an ingenious method.

## The equivalence of $(\phi ,\varphi )$-contractive and $(\phi ,\psi )$-contractive

We denote by $\Omega _1$ the class of all continuous non-decreasing functions $\varphi : (0,1]\rightarrow (0,1]$ such that $\lim _{t\rightarrow 0}\varphi (t)=0$ and $\varphi (1)=1$. We denote by $\Omega _2$ the class of all continuous non-decreasing functions $\psi : [0,+\infty )\rightarrow [0,+\infty )$ such that $\psi (0)=0$ and $\lim _{t\rightarrow +\infty }\varphi (t)=+\infty$. Further we give the following definition.

### **Definition 6**

Let (*E*, *F*, *T*) be a Menger probabilistic space and $f : E \rightarrow E$ be a mapping satisfying the following inequality

1 $$F_{fx,fy}(\phi (ct))\ge \varphi (F_{x,y}(\phi (t))) \quad \forall x,y \in E, \; \forall t>0,$$

where $c \in (0,1), \quad \phi \in \Phi , \; \varphi \in \Omega _1$. The mapping *f* satisfying condition (1) is called $(\phi ,\varphi )$-contractive mapping.

### **Definition 7**

Let (*E*, *F*, *T*) be a Menger probabilistic space and $f:E \rightarrow E$ be a mapping satisfying the following inequality

2 $$\frac{1}{F_{fx,fy}(\phi (ct))}-1\le \psi \left( \frac{1}{F_{x,y}(\phi (t))}-1\right) , \quad \forall x,y \in E, \; \forall t>0,$$

where $c \in (0,1), \phi \in \Phi , \quad \psi \in \Omega _2$. The mapping *f* satisfying condition (2) is called $(\phi ,\psi )$-contractive mapping.

### **Theorem 8**

*LetTbe a*$(\phi ,\psi )$-*contractive mapping, thenTis also a*$(\phi ,\varphi )$-*contractive mapping, where*

$$\varphi (t)=\frac{1}{\psi \left( \frac{1}{t}-1\right)+1}, \quad \ 0<t\le 1.$$

### *Proof*

We rewrite the (2) to the following form

$$\frac{1}{F_{fx,fy}(\phi (ct))}\le \psi \left( \frac{1}{F_{x,y}(\phi (t))}-1\right) +1, \quad \forall x,y \in E, \forall t>0,$$

which can be rewritten to

$$F_{fx,fy}(\phi (ct))\ge \frac{1}{\psi \left( \frac{1}{F_{x,y}(\phi (t))}-1\right) +1} , \quad \forall x,y \in E, \; \forall t>0.$$

That is

$$F_{fx,fy}(\phi (ct))\ge \varphi (F_{x,y}(\phi (t))) \quad \forall x,y \in E, \;\forall t>0.$$

This completes the proof. $\square$

### **Theorem 9**

*LetTbe a*$(\phi ,\varphi )$-*contractive mapping, thenTis also a*$(\phi ,\psi )$-*contractive mapping, where*

3 $$\psi (t)=\frac{1}{\varphi \left(\frac{1}{t+1}\right)} -1, \quad \ 0 \le t <+\infty .$$

### *Proof*

From the (3), we have

$$\varphi (t)=\frac{1}{\psi \left( \frac{1}{t}-1\right)+1}, \quad \ 0<t\le 1.$$

We rewrite the (1) to the following form

$$F_{fx,fy}(\phi (ct))\ge \frac{1}{\psi \left( \frac{1}{F_{x,y}(\phi (t))}-1\right)+1}, \quad \forall x,y \in E, \; \forall t>0,$$

which can be rewritten to

$$\frac{1}{F_{fx,fy}(\phi (ct))}\le \psi \left( \frac{1}{F_{x,y}(\phi (t))}-1\right) +1, \quad \forall x,y \in E, \; \forall t>0.$$

That is,

$$\frac{1}{F_{fx,fy}(\phi (ct))}-1\le \psi \left( \frac{1}{F_{x,y}(\phi (t))}-1\right) , \quad \forall x,y \in E, \; \forall t>0.$$

This completes the proof. $\square$

In this paper, we prove the following contraction mapping principle for the $(\phi ,\varphi )$-contractive mappings in a *G*-complete Menger probabilistic space. Meanwhile, we do not need to add the uniqueness condition of fixed point (see Kutbi et al. 2015).

### **Theorem 10**

*Let* (*E*, *F*, *T*) *be aG*-*complete Menger probabilistic space and*$f : E \rightarrow E$*be a*$(\phi ,\varphi )$-*contractive mapping. Assume that*$\lim _{a_n\rightarrow 1}\varphi ^n (a_n)=1$. *Thenfhas a unique fixed point.*

### *Proof*

For any $x_0 \in E$, we define a sequence $\{x_n\}$ by $x_{n+1}=Tx_n$ for all $n\ge 0$. From (1) and the properties of $\phi$ and $\varphi$ we know, for all $t>0$, that

4 $$\begin{aligned} F_{x_{n+1},x_n}(\phi (t)) &\ge \varphi \left(F_{x_n,x_{n-1}}\left(\phi \left(\frac{t}{c}\right)\right)\right)\ge \varphi \left( \varphi \left(F_{x_{n-1},x_{n-2}}\left(\phi \left(\frac{t}{c^2}\right)\right)\right)\right)\nonumber \\ &\ge \varphi ^3 \left(F_{x_{n-2},x_{n-3}}\left(\phi \left(\frac{t}{c^3}\right)\right)\right) \ge \cdots \ge \varphi ^n \left(F_{x_{1},x_{0}}\left(\phi \left(\frac{t}{c^n}\right)\right)\right)\rightarrow 1 \end{aligned}$$

as $n\rightarrow \infty$. Let $\varepsilon >0$ be given, then by using the properties (i) and (iv) of a function $\phi$ we can find $t>0$ such that $\varepsilon > \phi (t)$. It follows from (4) that

5 $$\lim _{n\rightarrow \infty }F_{x_{n+1},x_n}(\varepsilon )=1.$$

By using the triangle inequality (MPM-3), we obtain

$$\begin{aligned} F_{x_{n},x_{n+p}}(\varepsilon ) \ge T\left( F_{x_n,x_{n+1}}\left( \frac{\varepsilon }{p}\right) , T\left( F_{x_{n+1},x_{n+1}}\left( \frac{\varepsilon }{p}\right) , \ldots , \left( F_{x_{n+p-1},x_{n+p}}\left( \frac{\varepsilon }{p}\right) \right) \cdots \right) \right) . \end{aligned}$$

Thus, letting $n\rightarrow \infty$ and making use of (5), for any integer *p*, we get

$$\lim _{n\rightarrow \infty }F_{x_{n+p},x_n}(\varepsilon )=1, \quad \forall \varepsilon >0.$$

Hence $\{x_n\}$ is a *G*-Cauchy sequence. Since (*E*, *F*, *T*) is *G*-complete, there exists a point $u\in E$ such that $x_n\rightarrow u$ as $n\rightarrow \infty$. For any $\varepsilon >0$, choose $\phi (t) < \frac{\varepsilon }{2}$, we have

$$\begin{aligned} F_{fu,u}(\varepsilon ) &\ge T\left( F_{fu,x_{n+1}}\left( \frac{\varepsilon }{2}\right) , F_{x_{n+1},u}\left( \frac{\varepsilon }{2}\right) \right) ,\\ &\ge T\left(F_{fu,x_{n+1}}(\phi (t)), F_{x_{n+1},u}\left( \frac{\varepsilon }{2}\right) \right),\\ &\ge T\left( F_{u,x_{n}}\left(\phi \left(\frac{t}{c}\right)\right), F_{x_{n+1},u}\left( \frac{\varepsilon }{2}\right) \right) \rightarrow 1, \end{aligned}$$

as $n\rightarrow \infty$, which in turn yields that $fu=u$. Next we show the uniqueness of the fixed point. If there exists *v* such that $fv=v$, by using (3) we hvae

$$F_{u,v}(\phi (t))= F_{fu,fv}(\phi (t))\ge \varphi \left(F_{u,v}\left(\phi \left(\frac{t}{c}\right)\right)\right)\ge \cdots \ge \varphi ^n \left( F_{u,v}\left( \phi \left( \frac{t}{c^n}\right) \right) \right) \rightarrow 1$$

as $n\rightarrow \infty$. It is easy to see $u=v$. The proof is completed. $\square$

Kutbi et al. (2015) proved the following fixed point theorem for the $(\phi ,\psi )$-contractive mappings in a *G*-complete Menger probabilistic space. Meanwhile, they need to add the uniqueness condition of fixed point (see Xu et al. 2015). In order to clearly show the content of theorem, we use a clear form to write this theorem.

### **Theorem 11**

(Kutbi et al. 2015) *Let* (*E*, *F*, *T*) *be aG*-*complete Menger probabilistic space and*$f : E \rightarrow E$*be a*$(\phi ,\psi )$-*contractive mapping. Assume that*$\lim _{a_n\rightarrow 0}\psi ^n (a_n)=0$. *Thenfhas a fixed point.*

In order to get the uniqueness of fixed point, authors added the following condition:

$$F_{u,v}(0)=0, \quad \forall u, v \in F(f), \quad\quad\quad\quad\quad (*)$$

where *F*(*f*) denotes the set of all fixed points of a mapping *f*.

### **Theorem 12**

(Kutbi et al. 2015) *Adding condition*$(*)$*to the hypotheses ofTheorem* 11, *we obtain uniqueness of the fixed point.*

By using Theorem 10, we can get the following contraction mapping principle for the $(\phi ,\psi )$-contractive mappings in a *G*-complete Menger probabilistic space.

### **Theorem 13**

*Let* (*E*, *F*, *T*) *be aG*-*complete Menger probabilistic space and*$f : E \rightarrow E$*be a*$(\phi ,\psi )$-*contractive mapping. Assume that*$\lim _{a_n\rightarrow 1}\varphi ^n (a_n)=1$. *Thenfhas a unique fixed point, where*

$$\varphi (t)=\frac{1}{\psi ( \frac{1}{t}-1)+1}, \quad 0<t\le 1.$$

### *Proof*

From Theorem 8, we know that, *T* is also a $(\phi ,\varphi )$-contractive mapping, where

$$\varphi (t)=\frac{1}{\psi ( \frac{1}{t}-1)+1}, \quad 0<t\le 1.$$

Since $\lim _{a_n\rightarrow 1}\varphi ^n (a_n)=1$, by using Theorem 8, we obtain the conclusion. This completes the proof. $\square$

### **Open question 14**

Is the following property right?

6 $$\lim _{b_n\rightarrow 1}\varphi ^n (b_n)=1 \Leftrightarrow \lim _{a_n\rightarrow 0}\psi ^n (a_n)=0,$$

where

$$\varphi (t)=\frac{1}{\psi ( \frac{1}{t}-1)+1}, \quad 0<t\le 1.$$

If the property (6) is right, then we can obtain the following result.

### **Theorem 15**

*Let* (*E*, *F*, *T*) *be aG*-*complete Menger probabilistic space and*$f : E \rightarrow E$*be a*$(\phi ,\psi )$-*contractive mapping. Assume that*$\lim _{a_n\rightarrow 0}\psi ^n (a_n)=0$. *Thenfhas a unique fixed point.*

### **Conclusion 16**

The property (6) is right. Therefore Theorem 15 holds.

### *Proof*

It is not hard to show that, the property (6) is equivalent to the following proposition

7 $$\lim _{b_n\rightarrow 1}\varphi ^n (b_n)=1 \Leftrightarrow \lim _{a_n\rightarrow 0}\psi ^n (a_n)=0,$$

where $$a_n=\frac{1}{b_n}-1$$ and

$$\varphi (t)=\frac{1}{\psi ( \frac{1}{t}-1)+1}, \quad 0<t\le 1.$$

Next, we prove (7). Let

$$B(t)=\frac{1}{1+t},\quad A(t)=\frac{1}{t}-1,\quad 0<t\le 1,$$

then we have

$$\begin{aligned}&B^{-1}=A,\quad A^{-1}=B,\\ &a_n=\frac{1}{b_n}-1=A(b_n),\quad b_n=\frac{1}{a_n+1}=B(a_n),\\&\varphi (t)=\frac{1}{\psi ( \frac{1}{t}-1)+1}=\frac{1}{\psi (A(t))+1}=B\psi A(t), \quad 0<t\le 1. \\&\psi (t)=A\varphi B(t), \quad 0\le t<+\infty . \end{aligned}$$

Now we prove

$$\begin{aligned} \lim _{b_n\rightarrow 1}\varphi ^n (b_n)=1 \Rightarrow \lim _{a_n\rightarrow 0}\psi ^n (a_n)=0. \end{aligned}$$

Observe

$$\begin{aligned} \psi ^n(a_n)&=\psi ^{n-1}\psi (a_n) \\&= \psi ^{n-1}A\varphi B(a_n) \\&= \psi ^{n-2}A\varphi BA\varphi B(a_n) \\&= \dots \\&= (A\varphi B)^n(a_n) \\&= (A\varphi B)^nA(b_n) \\&= A\varphi ^n(b_n). \end{aligned}$$

Because $\lim _{n\rightarrow \infty }\varphi ^n (b_n)= 1$ and $\lim _{t\rightarrow 1}A(t)=0$, we have $\lim _{a_n\rightarrow 0}\psi ^n (a_n)=0$.

Now we prove

$$\lim _{b_n\rightarrow 1}\varphi ^n (b_n)=1 \Leftarrow \lim _{a_n\rightarrow 0}\psi ^n (a_n)=0.$$

Observe

$$\begin{aligned} \varphi ^n(b_n)&=\varphi ^{n-1}\varphi (b_n) \\&=\varphi ^{n-1}B\psi A(b_n) \\&=\varphi ^{n-2}(B\psi A)^2(b_n) \\&=\dots \\&=(B\varphi A)^n(b_n) \\&=(B\varphi A)^nB(a_n) \\&=B\psi ^n(a_n) \end{aligned}$$

Because $\lim _{n\rightarrow \infty }\psi ^n (a_n)= 0$ and $\lim _{t\rightarrow 0}B(t)=1$, we have $\lim _{b_n\rightarrow 1}\varphi ^n (b_n)=1$.

This completes the proof. $\square$

## Examples

### **Theorem 17**

*Let* (*X*, *d*) *be a metric space,*$f: X\rightarrow X$*be a mapping satisfying the following condition:*

8 $$d(f(x),f(y))\le 2c^2d(x,y), \quad \forall x,y \in X,$$

*where*$c \in (0,1)$*is a constant. Let*

$$F_{x,y}(t)={\left\{ \begin{array}{ll} \frac{t}{t+d(x,y)},& \quad t>0,\\ 0,& \quad t\le 0, \end{array}\right. } \quad \forall x,y \in X.$$

*Then*

1. $(X,F,T_4)$*is a Menger probabilistic metric space;*
2. *Tis a*$(\phi ,\varphi )$-*contractive mapping, where*$\phi (t)=\varphi (t)=t^2$;
3. *Tis also a*$(\phi ,\psi )$-*contractive mapping, where*$\phi (t)=t^2, \psi (t)=t^2+2t$.

### *Proof*

(1) We prove $(X,F,T_4)$ is a Menger probabilistic metric space. The conditions (MPM-1) and (MPM-2) obviously hold. We prove the condition (MPM-3). For any $x,y,z \in X$ and $t>0, s>0$, we claim that

$$\frac{t+s}{(t+s)+d(x,y)}\ge \min \left\{ \frac{t}{t+d(x,z)},\quad \frac{s}{s+d(z,y)}\right\} .$$

If not, we have

$$\begin{aligned} \frac{t+s}{(t+s)+d(x,y)} &< \frac{t}{t+d(x,z)},\\ \frac{t+s}{(t+s)+d(x,y)} &< \frac{s}{s+d(z,y)} \end{aligned}$$

which is equivalent to

$$\begin{aligned} (t+s)(t+d(x,z)) &< t((t+s)+d(x,y)),\\ (t+s)(s+d(z,y)) &< s((t+s)+d(x,y)). \end{aligned}$$

Adding the above two inequalities, we get

$$(t+s)(d(x,z)+d(z,y))<(t+s)d(x,y)$$

which implies

$$d(x,z)+d(z,y)<d(x,y).$$

This is a contradiction which implies the condition (MPM-3) holds.

(2) From (8) we have

$$d(f(x),f(y))\le (2c^2+\frac{c^2}{t^2}d(x,y))d(x,y), \quad \forall x,y \in X, \ t>0,$$

and hence

9 $$\begin{aligned} &d(f(x),f(y)) \le \frac{1}{t^2}\left( 2c^2t^2+c^2d(x,y)\right) d(x,y),\quad \forall x,y \in X, \; t>0,\nonumber \\ & t^2d(f(x),f(y)) \le (2c^2t^2+c^2d(x,y))d(x,y),\quad \forall x,y \in X, \; t>0,\nonumber \\ &c^2t^4+t^2d(f(x),f(y)) \le c^2t^4+(2c^2t^2+c^2d(x,y))d(x,y),\quad \forall x,y \in X, \; t>0,\nonumber \\ &t^2(c^2t^2+d(f(x),f(y))\le c^2(t^4+2t^2d(x,y)+d^2(x,y)),\quad \forall x,y \in X, \; t>0,\nonumber \\ &t^2(c^2t^2+d(f(x),f(y)))\le c^2(t^2+d(x,y))^2, \quad \forall x,y \in X, \; t>0,\nonumber \\ &\frac{t^4}{(t^2+d(x,y))^2}\le \frac{c^2t^2}{c^2t^2+d(f(x),f(y))},\quad \forall x,y \in X, \; t>0,\nonumber \\ &\left(\frac{t^2}{t^2+d(x,y)}\right)^2\le \frac{c^2t^2}{c^2t^2+d(f(x),f(y))},\quad \forall x,y \in X, \; t>0. \end{aligned}$$

We rewrite inequality (9) to the following form

$$F_{f(x),f(y)}((ct)^2)\ge (F_{x,y}(t^2))^2,\quad \forall x,y \in X,\; t>0.$$

That is,

$$F_{f(x),f(y)}(\phi (ct))\ge \varphi (F_{x,y}(\phi (t)),\quad \forall x,y \in X, \; t>0,$$

where $$\phi (t)=t^2, \varphi (t)=t^2.$$

(3) By using Theorem 9, we know that, *T* is also a $(\phi ,\psi )$-contractive mapping with

$$\psi (t)=\frac{1}{\varphi (\frac{1}{t+1})} -1, \quad 0 \le t < +\infty .$$

That is $\psi (t)=\frac{1}{ (\frac{1}{t+1})^2}-1=t^2+t$. This completes the proof. $\square$

### **Theorem 18**

*Let* (*X*, *d*) *be a metric space,*$f: X\rightarrow X$*be a nonexpansive mapping. Let*

$$F_{x,y}(t)={\left\{ \begin{array}{ll} \frac{t}{t+d(x,y)}, &\quad t>0,\\ 0, &\quad t\le 0, \end{array}\right. } \quad \forall x,y \in X.$$

Then

1. $(X,F,T_4)$*is a Menger probabilistic metric space;*
2. *Tis a*$(\phi ,\varphi )$-*contractive mapping, where*$\phi (t)=t^2, \varphi (t)=\frac{(1+t)t}{2}$;
3. *Tis also a*$(\phi ,\psi )$-*contractive mapping, where*$\phi (t)=t^2,\quad \psi (t)=\frac{2t^2+3t}{2+t}$.

### *Proof*

(1) It is a conclusion of Theorem 17. (2) Since *T* is nonexpansive, let $c \in (0,1)$ be a constant such that $3c^2\ge 2$, we have

$$d(f(x),f(y))\le \frac{3c^2t^2+d(x,y)}{2t^2+d(x,y)}d(x,y), \quad \forall x,y \in X, \quad t>0,$$

and hence

10 $$\begin{aligned} &(2t^2+d(x,y))d(f(x),f(y)) \le (3c^2t^2+d(x,y))d(x,y), \quad \forall x,y \in X, \; t>0. \nonumber \\&2c^2t^4+2t^2d(f(x),f(y))+c^2t^2d(x,y)+d(f(x),f(y))d(x,y) \\&\quad\le 2c^2t^4+4c^2t^2d(x,y)+(d(x,y))^2, \quad \forall x,y \in X, \; t>0. \nonumber \\ &(c^2t^2+d(f(x),f(y)))(2t^2+d(x,y)) \\&\quad\le 2c^2t^4+4c^2t^2d(x,y)+(d(x,y))^2, \quad \forall x,y \in X, \quad t>0.\nonumber \\ &\frac{2t^2+d(x,y)}{(t^2+d(x,y))^2} \le \frac{2c^2}{c^2t^2+d(f(x),f(y))}, \quad \forall x,y \in X, \; t>0.\nonumber \\ &\left(1+\frac{t^2}{t^2+d(x,y)}\right)\frac{t^2}{t^2+d(x,y} \le \frac{2c^2t^2}{c^2t^2+d(f(x),f(y))}, \quad \forall x,y \in X, \; t>0.\nonumber \\ &\frac{1}{2}\left(1+\frac{t^2}{t^2+d(x,y)}\right)\frac{t^2}{t^2+d(x,y)} \le \frac{c^2t^2}{c^2t^2+d(f(x),f(y))}, \quad \forall x,y \in X, \; t>0. \end{aligned}$$

We rewrite inequality (10) to the following form

$$F_{f(x),f(y)}((ct)^2)\ge \frac{1+F_{x,y}(t^2)}{2}F_{x,y}(t^2), \quad \forall x,y \in X, \; t>0.$$

That is,

$$F_{f(x),f(y)}(\phi (ct))\ge \varphi (F_{x,y}(\phi (t)),\quad \forall x,y \in X, \; t>0.$$

where $\phi (t)=t^2, \quad \varphi (t)=\frac{(1+t)t}{2}.$ (3) By using Theorem 9, we know that, *T* is also a $(\phi ,\psi )$-contractive mapping with

$$\psi (t)=\frac{1}{\varphi (\frac{1}{t+1})} -1, \quad 0 \le t < +\infty .$$

That is,

$$\begin{aligned} \psi (t)&=\frac{2}{ (1+\frac{1}{1+t})\frac{1}{1+t}}-1\\&=\frac{2(1+t)^2}{2+t}-1\\&=\frac{2t^2+3t}{2+t}. \end{aligned}$$

This completes the proof. $\square$
